# 2′-(3-Bromo-5-chloro-2-hydroxy­benzyl­idene)isonicotinohydrazide methanol solvate

**DOI:** 10.1107/S1600536808019648

**Published:** 2008-07-05

**Authors:** Chun-Bao Tang

**Affiliations:** a Department of Chemistry, Jiaying University, Meizhou 514015, People’s Republic of China

## Abstract

The title Schiff base compound, C_13_H_9_BrClN_3_O_2_·CH_4_O, was derived from the condensation reaction of 3-bromo-5-chloro­salicylaldehyde with isonicotinohydrazide. The dihedral angle between the benzene and pyridine rings is 5.9 (2)°. In the crystal structure, mol­ecules are linked through N—H⋯O, O—H⋯O, and O—H⋯Br inter­molecular hydrogen bonds, forming dimers and chains. There is also an intramolecular O—H⋯N hydrogen bond.

## Related literature

For related structures, see: Tang, (2006 ▶); Tang, (2007*a* ▶,*b* ▶,*c* ▶,*d* ▶). For reference structural data, see: Allen *et al.* (1987 ▶).
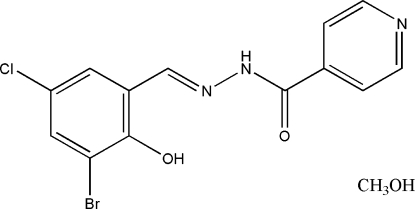


## Experimental

### 

#### Crystal data

C_13_H_9_BrClN_3_O_2_·CH_4_O
*M* *_r_* = 386.63
Triclinic, 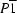

*a* = 7.531 (1) Å
*b* = 8.735 (1) Å
*c* = 12.130 (2) Å
α = 80.853 (2)°
β = 77.781 (2)°
γ = 86.721 (2)°
*V* = 769.73 (19) Å^3^
*Z* = 2
Mo *K*α radiation
μ = 2.86 mm^−1^
*T* = 298 (2) K
0.32 × 0.32 × 0.30 mm

#### Data collection

Bruker SMART CCD area-detector diffractometer
Absorption correction: multi-scan (*SADABS*; Sheldrick, 1996 ▶) *T*_min_ = 0.461, *T*_max_ = 0.481 (expected range = 0.407–0.424)
4529 measured reflections
3254 independent reflections
2438 reflections with *I* > 2σ(*I*)
*R*_int_ = 0.018

#### Refinement

*R*[*F* ^2^ > 2σ(*F* ^2^)] = 0.039
*wR*(*F* ^2^) = 0.093
*S* = 1.01
3254 reflections
205 parameters
1 restraint
H atoms treated by a mixture of independent and constrained refinement
Δρ_max_ = 0.44 e Å^−3^
Δρ_min_ = −0.36 e Å^−3^

### 

Data collection: *SMART* (Bruker, 2002 ▶); cell refinement: *SAINT* (Bruker, 2002 ▶); data reduction: *SAINT*; program(s) used to solve structure: *SHELXS97* (Sheldrick, 2008 ▶); program(s) used to refine structure: *SHELXL97* (Sheldrick, 2008 ▶); molecular graphics: *SHELXTL* (Sheldrick, 2008 ▶); software used to prepare material for publication: *SHELXL97*.

## Supplementary Material

Crystal structure: contains datablocks global, I. DOI: 10.1107/S1600536808019648/at2582sup1.cif

Structure factors: contains datablocks I. DOI: 10.1107/S1600536808019648/at2582Isup2.hkl

Additional supplementary materials:  crystallographic information; 3D view; checkCIF report

## Figures and Tables

**Table 1:** Hydrogen-bond geometry (Å, °)

*D*—H⋯*A*	*D*—H	H⋯*A*	*D*⋯*A*	*D*—H⋯*A*
N2—H2⋯O3^i^	0.890 (10)	2.005 (15)	2.876 (4)	166 (4)
O3—H3⋯Br1	0.82	3.05	3.641 (3)	131
O3—H3⋯O1	0.82	2.62	3.268 (4)	137
O3—H3⋯O1^ii^	0.82	2.55	3.114 (4)	127
O1—H1⋯N1	0.82	1.87	2.590 (3)	146

Symmetry codes: (i) 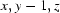; (ii) 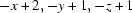.

**Fig. 1.**
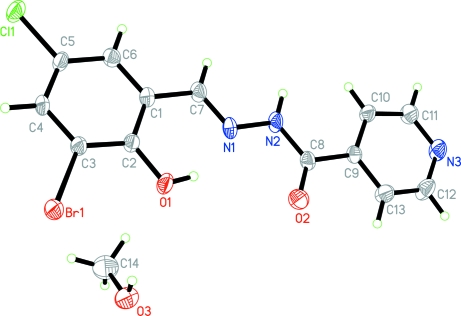
The molecular structure of the compound, showing the atom-numbering scheme. Displacement ellipsoids are drawn at the 30% probability level.

**Fig. 2.**
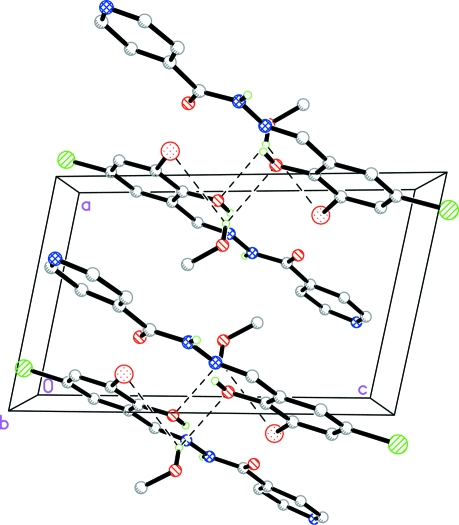
Molecular packing of (I) with hydrogen bonds drawn as dashed lines.

## supplementary crystallographic information

### Comment

Recently, the author has reported the structures of several Schiff base compounds (Tang, 2006; Tang, 2007*a*,b,c,d) and, in continuation of work in this area, reports herein the crystal structure of the title new Schiff base compound, (I).

In the title compound (Fig. 1), the dihedral angle between the benzene ring and the pyridine ring is 5.9 (2)°. The torsion angles C1—C7—N1—N2, C7—N1—N2—C8, and N1—N2—C8—C9 are 0.4 (2), 2.3 (2), and 1.9 (2)°, respectively. All the bond lengths are within normal values (Allen *et al.*, 1987).

In the crystal structure of the compound, molecules are linked through N–H···O, O–H···O, and O–H···Br intermolecular hydrogen bonds (Table 1), forming dimers (Fig. 2).

### Experimental

3-Bromo-5-chlorosalicylaldehyde (0.1 mmol, 23.5 mg) and isonicotinohydrazide (0.1 mmol, 13.7 mg) were dissolved in a methanol solution (20 ml). The mixture was stirred at reflux for 10 min to give a clear colourless solution. Colourless block-like crystals of the compound were formed by slow evaporation of the solvent over several days.

### Refinement

H2 was located from a difference Fourier map and refined isotropically, with *U*_iso_ restrained to 0.08Å^2^. Other H atoms were constrained to ideal geometries, with d(C–H) = 0.93–0.96 Å, d(O–H) = 0.82 Å, and with *U*_iso_(H) = 1.2*U*_eq_(C), 1.5*U*_eq_(C14, O1 and O3).

### Crystal data

**Table:**

C_13_H_9_BrClN_3_O_2_·C_1_H_4_O_1_	*Z* = 2
*M**_r_* = 386.63	*F*_000_ = 388
Triclinic, *P*1	*D*_x_ = 1.668 Mg m^−^^3^
Hall symbol: -P 1	Mo *K*α radiation λ = 0.71073 Å
*a* = 7.531 (1) Å	Cell parameters from 1404 reflections
*b* = 8.735 (1) Å	θ = 2.5–24.3º
*c* = 12.130 (2) Å	µ = 2.86 mm^−^^1^
α = 80.853 (2)º	*T* = 298 (2) K
β = 77.781 (2)º	Block, colourless
γ = 86.721 (2)º	0.32 × 0.32 × 0.30 mm
*V* = 769.73 (19) Å^3^	

### Data collection

**Table:**

Bruker SMART CCD area-detector diffractometer	3254 independent reflections
Radiation source: fine-focus sealed tube	2438 reflections with *I* > 2σ(*I*)
Monochromator: graphite	*R*_int_ = 0.018
*T* = 298(2) K	θ_max_ = 27.0º
ω scans	θ_min_ = 2.4º
Absorption correction: multi-scan(SADABS; Sheldrick, 1996)	*h* = −9→9
*T*_min_ = 0.461, *T*_max_ = 0.481	*k* = −11→10
4529 measured reflections	*l* = −15→13

### Refinement

**Table:**

Refinement on *F*^2^	Secondary atom site location: difference Fourier map
Least-squares matrix: full	Hydrogen site location: inferred from neighbouring sites
*R*[*F*^2^ > 2σ(*F*^2^)] = 0.039	H atoms treated by a mixture of independent and constrained refinement
*wR*(*F*^2^) = 0.093	*w* = 1/[σ^2^(*F*_o_^2^) + (0.0424*P*)^2^ + 0.2117*P*] where *P* = (*F*_o_^2^ + 2*F*_c_^2^)/3
*S* = 1.01	(Δ/σ)_max_ < 0.001
3254 reflections	Δρ_max_ = 0.44 e Å^−^^3^
205 parameters	Δρ_min_ = −0.36 e Å^−^^3^
1 restraint	Extinction correction: none
Primary atom site location: structure-invariant direct methods	

### Special details

**Table:**

Geometry. All esds (except the esd in the dihedral angle between two l.s. planes) are estimated using the full covariance matrix. The cell esds are taken into account individually in the estimation of esds in distances, angles and torsion angles; correlations between esds in cell parameters are only used when they are defined by crystal symmetry. An approximate (isotropic) treatment of cell esds is used for estimating esds involving l.s. planes.
Refinement. Refinement of F^2^ against ALL reflections. The weighted R-factor wR and goodness of fit S are based on F^2^, conventional R-factors R are based on F, with F set to zero for negative F^2^. The threshold expression of F^2^ > 2sigma(F^2^) is used only for calculating R-factors(gt) etc. and is not relevant to the choice of reflections for refinement. R-factors based on F^2^ are statistically about twice as large as those based on F, and R- factors based on ALL data will be even larger.

### Fractional atomic coordinates and isotropic or equivalent isotropic displacement parameters (Å^2^)

**Table:**

	*x*	*y*	*z*	*U*_iso_*/*U*_eq_	
Br1	1.13987 (5)	0.60600 (4)	0.27243 (3)	0.04953 (14)	
Cl1	1.14281 (14)	0.21720 (11)	−0.04331 (7)	0.0585 (3)	
O1	0.9336 (4)	0.3429 (3)	0.42960 (18)	0.0492 (6)	
H1	0.8805	0.2694	0.4718	0.074*	
O2	0.6850 (4)	0.1108 (3)	0.6960 (2)	0.0613 (7)	
O3	0.7162 (4)	0.6702 (3)	0.4708 (2)	0.0648 (7)	
H3	0.8129	0.6213	0.4678	0.097*	
N1	0.7910 (4)	0.0712 (3)	0.4821 (2)	0.0422 (7)	
N2	0.7009 (4)	−0.0439 (3)	0.5622 (2)	0.0435 (7)	
N3	0.3580 (4)	−0.3481 (4)	0.9325 (3)	0.0541 (8)	
C1	0.9409 (4)	0.1661 (4)	0.2938 (3)	0.0376 (7)	
C2	0.9789 (4)	0.3099 (3)	0.3219 (2)	0.0356 (7)	
C3	1.0695 (4)	0.4184 (3)	0.2357 (3)	0.0371 (7)	
C4	1.1193 (4)	0.3917 (3)	0.1240 (3)	0.0383 (7)	
H4	1.1788	0.4670	0.0674	0.046*	
C5	1.0792 (4)	0.2518 (4)	0.0983 (3)	0.0388 (7)	
C6	0.9935 (4)	0.1387 (4)	0.1807 (3)	0.0406 (8)	
H6	0.9703	0.0439	0.1615	0.049*	
C7	0.8466 (4)	0.0454 (4)	0.3797 (3)	0.0423 (8)	
H7	0.8271	−0.0499	0.3599	0.051*	
C8	0.6508 (4)	−0.0112 (4)	0.6708 (3)	0.0392 (7)	
C9	0.5474 (4)	−0.1341 (3)	0.7577 (3)	0.0353 (7)	
C10	0.5137 (5)	−0.2802 (4)	0.7393 (3)	0.0432 (8)	
H10	0.5524	−0.3098	0.6675	0.052*	
C11	0.4218 (5)	−0.3815 (4)	0.8288 (3)	0.0533 (9)	
H11	0.4032	−0.4806	0.8155	0.064*	
C12	0.3884 (5)	−0.2064 (5)	0.9486 (3)	0.0601 (10)	
H12	0.3433	−0.1788	1.0204	0.072*	
C13	0.4825 (5)	−0.0977 (4)	0.8658 (3)	0.0507 (9)	
H13	0.5023	−0.0005	0.8825	0.061*	
C14	0.6322 (7)	0.6375 (5)	0.3861 (4)	0.0809 (14)	
H14A	0.5114	0.6820	0.3967	0.121*	
H14B	0.6264	0.5272	0.3902	0.121*	
H14C	0.7007	0.6808	0.3127	0.121*	
H2	0.689 (6)	−0.136 (2)	0.543 (3)	0.080*	

### Atomic displacement parameters (Å^2^)

**Table:**

	*U*^11^	*U*^22^	*U*^33^	*U*^12^	*U*^13^	*U*^23^
Br1	0.0660 (3)	0.0388 (2)	0.0409 (2)	−0.01820 (16)	0.00010 (16)	−0.00617 (14)
Cl1	0.0847 (7)	0.0584 (5)	0.0301 (4)	−0.0171 (5)	0.0016 (4)	−0.0118 (4)
O1	0.0672 (17)	0.0464 (14)	0.0289 (12)	−0.0191 (12)	0.0035 (11)	−0.0019 (10)
O2	0.088 (2)	0.0386 (13)	0.0523 (15)	−0.0236 (13)	0.0019 (13)	−0.0050 (11)
O3	0.073 (2)	0.0651 (18)	0.0574 (17)	−0.0025 (15)	−0.0088 (15)	−0.0168 (14)
N1	0.0448 (16)	0.0384 (15)	0.0373 (16)	−0.0112 (12)	−0.0042 (12)	0.0104 (12)
N2	0.0547 (18)	0.0384 (15)	0.0331 (15)	−0.0216 (14)	−0.0030 (13)	0.0063 (12)
N3	0.059 (2)	0.0546 (19)	0.0415 (17)	−0.0192 (15)	0.0025 (14)	0.0047 (14)
C1	0.0376 (18)	0.0377 (17)	0.0344 (17)	−0.0055 (14)	−0.0053 (14)	0.0028 (13)
C2	0.0383 (18)	0.0375 (17)	0.0292 (16)	−0.0064 (14)	−0.0041 (13)	−0.0017 (13)
C3	0.0421 (19)	0.0339 (16)	0.0331 (16)	−0.0103 (14)	−0.0024 (14)	−0.0028 (13)
C4	0.0418 (19)	0.0351 (17)	0.0322 (16)	−0.0098 (14)	0.0003 (14)	0.0048 (13)
C5	0.0451 (19)	0.0439 (18)	0.0256 (15)	−0.0084 (15)	−0.0029 (14)	−0.0028 (13)
C6	0.046 (2)	0.0361 (17)	0.0405 (18)	−0.0097 (14)	−0.0095 (15)	−0.0049 (14)
C7	0.047 (2)	0.0376 (17)	0.0394 (19)	−0.0157 (15)	−0.0047 (15)	0.0022 (14)
C8	0.0424 (19)	0.0341 (17)	0.0374 (18)	−0.0089 (14)	−0.0042 (14)	0.0037 (14)
C9	0.0334 (17)	0.0346 (16)	0.0349 (17)	−0.0086 (13)	−0.0030 (13)	0.0014 (13)
C10	0.052 (2)	0.0387 (18)	0.0335 (17)	−0.0125 (15)	0.0030 (15)	−0.0025 (14)
C11	0.062 (2)	0.0433 (19)	0.049 (2)	−0.0178 (18)	0.0004 (18)	0.0006 (16)
C12	0.068 (3)	0.072 (3)	0.035 (2)	−0.022 (2)	0.0059 (18)	−0.0078 (18)
C13	0.061 (2)	0.0446 (19)	0.043 (2)	−0.0167 (17)	0.0042 (17)	−0.0090 (16)
C14	0.095 (4)	0.077 (3)	0.078 (3)	−0.012 (3)	−0.025 (3)	−0.019 (3)

### Geometric parameters (Å, °)

**Table:**

Br1—C3	1.897 (3)	C4—C5	1.373 (4)
Cl1—C5	1.753 (3)	C4—H4	0.9300
O1—C2	1.351 (3)	C5—C6	1.370 (4)
O1—H1	0.8200	C6—H6	0.9300
O2—C8	1.209 (4)	C7—H7	0.9300
O3—C14	1.388 (5)	C8—C9	1.503 (4)
O3—H3	0.8200	C9—C10	1.377 (4)
N1—C7	1.276 (4)	C9—C13	1.380 (4)
N1—N2	1.380 (3)	C10—C11	1.374 (4)
N2—C8	1.363 (4)	C10—H10	0.9300
N2—H2	0.890 (10)	C11—H11	0.9300
N3—C11	1.320 (5)	C12—C13	1.373 (5)
N3—C12	1.323 (5)	C12—H12	0.9300
C1—C6	1.400 (4)	C13—H13	0.9300
C1—C2	1.412 (4)	C14—H14A	0.9600
C1—C7	1.457 (4)	C14—H14B	0.9600
C2—C3	1.383 (4)	C14—H14C	0.9600
C3—C4	1.380 (4)		
			
C2—O1—H1	109.5	N1—C7—H7	120.2
C14—O3—H3	109.5	C1—C7—H7	120.2
C7—N1—N2	118.9 (3)	O2—C8—N2	122.1 (3)
C8—N2—N1	116.0 (3)	O2—C8—C9	121.6 (3)
C8—N2—H2	124 (3)	N2—C8—C9	116.3 (3)
N1—N2—H2	120 (3)	C10—C9—C13	117.2 (3)
C11—N3—C12	115.9 (3)	C10—C9—C8	125.7 (3)
C6—C1—C2	119.4 (3)	C13—C9—C8	117.0 (3)
C6—C1—C7	118.9 (3)	C11—C10—C9	118.7 (3)
C2—C1—C7	121.6 (3)	C11—C10—H10	120.6
O1—C2—C3	119.5 (3)	C9—C10—H10	120.6
O1—C2—C1	122.3 (3)	N3—C11—C10	124.7 (3)
C3—C2—C1	118.2 (3)	N3—C11—H11	117.6
C4—C3—C2	122.3 (3)	C10—C11—H11	117.6
C4—C3—Br1	118.4 (2)	N3—C12—C13	124.1 (3)
C2—C3—Br1	119.3 (2)	N3—C12—H12	118.0
C5—C4—C3	118.6 (3)	C13—C12—H12	118.0
C5—C4—H4	120.7	C12—C13—C9	119.3 (3)
C3—C4—H4	120.7	C12—C13—H13	120.4
C6—C5—C4	121.6 (3)	C9—C13—H13	120.4
C6—C5—Cl1	119.6 (2)	O3—C14—H14A	109.5
C4—C5—Cl1	118.8 (2)	O3—C14—H14B	109.5
C5—C6—C1	119.9 (3)	H14A—C14—H14B	109.5
C5—C6—H6	120.1	O3—C14—H14C	109.5
C1—C6—H6	120.1	H14A—C14—H14C	109.5
N1—C7—C1	119.6 (3)	H14B—C14—H14C	109.5

### Hydrogen-bond geometry (Å, °)

**Table:**

*D*—H···*A*	*D*—H	H···*A*	*D*···*A*	*D*—H···*A*
N2—H2···O3^i^	0.890 (10)	2.005 (15)	2.876 (4)	166 (4)
O3—H3···Br1	0.82	3.05	3.641 (3)	131
O3—H3···O1	0.82	2.62	3.268 (4)	137
O3—H3···O1^ii^	0.82	2.55	3.114 (4)	127
O1—H1···N1	0.82	1.87	2.590 (3)	146

Symmetry codes: (i) *x*, *y*−1, *z*; (ii) −*x*+2, −*y*+1, −*z*+1.
